# Supplementary material for: Unmasking individual differences in adult reading procedures by disrupting holistic orthographic perception
Source: PLoS One. 2020 May 26;15(5):e0233041. doi: 10.1371/journal.pone.0233041 (PMC7250424; doi:10.1371/journal.pone.0233041)
Supplement: Attachment — Submitted filename: ResponseToReviewers_Final2.docx [file pone.0233041.s003.docx]

Dear Dr. Yafit Gabay and Reviewers,

We very much appreciate the helpful comments and believe they have greatly improved the paper. Responses to individual comments are below in blue italics.

Sincerely,

Elizabeth Hirshorn, PhD

Reviewer #1: OVERALL IMPRESSION:

My overall impression is somewhat positive re. the study overall but I am worried about the possibility of low power. I think that the main idea is interesting, and it seems that the study was well executed. Sample size is small (N = 14 and N = 11) which made me question the replicability of certain outcomes, both positive and negative. In any case, a small sample makes it particularly important IMO to show the underlying data points explicitly and graphically in the manuscript.

*We appreciate this point and have included figures for the most critical results of the paper (see new Fig 2 and 3). We understand the concern of small sample size and have made a point to be transparent in describing strong results that are statistically unlikely due to chance, and versus those that are more tentative. We have also added text to our discussion section (pgs. 23 and 27) that explicitly notes our relatively low sample size and that recommends future work to test whether the results generalize to other groups.*

COMMENTS AND SUGGESTIONS:

-- Introduction --

“An atypical orientation is thought to disproportionately affect holistically processed objects because the holistic process cannot be applied when the object is presented unconventionally.” While the former part is widely accepted, I wanted to point the authors to this counterargument to the latter part:

Richler, J. J., Mack, M. L., Palmeri, T. J., & Gauthier, I. (2011). Inverted faces are (eventually) processed holistically. Vision Research, 51(3), 333-342.

*Thank you for this point. We have added this reference to the introduction at the bottom of page 3 of the manuscript. We would argue that even if perception is ultimately holistic, inversion sensitivity could still be used as a marker of reading procedures.*

“Chinese is a morpho-syllabic writing system, and so sublexical orthographic coding and mapping to phonology is less useful for word identification, as compared to the holistic coding and mapping of characters to their morphemic forms.” Whether or not expert Chinese readers rely on holistic processing of Chinese characters might depend on their writing experience: “Compared with Chinese nonreaders, Chinese readers who had limited writing experience showed increased holistic processing, whereas Chinese readers who could write characters fluently showed reduced holistic processing.”

Tso, R. V. Y., Au, T. K. F., & Hsiao, J. H. W. (2014). Perceptual expertise: can sensorimotor experience change holistic processing and left-side bias? Psychological Science, 25(9), 1757-1767.

*This point is well taken. We have added this reference and made the point that the degree of holistic processing varies in Chinese readers, although Chinese readers may still on average rely more on holistic processing than alphabetic readers (see pg. 4-5 of manuscript).*

-- Materials and Methods –

---- Lexical decision and Overt word naming----

As already mentioned, the sample size was quite small. The initial 2AFC (I am assuming, not much info is given – I suggest giving more detail about tasks and stimuli, randomization/blocking etc.) lexical decision screening task also only included 40 trials, which might lead to unreliable RT estimates and therefore unreliable group membership assignment.

*This is correct. The initial lexical decision task was part of a larger screening battery, and therefore was meant to be very short. (In subsequent research, we have increased the number of trials.) More detail has been added to the methods on page 10 of the manuscript.*

I was therefore happy to see that the author reassessed orientation sensitivity. This manipulation is however different from the one used in lexical decision. In lexical decision, parts and wholes were both inverted (akin to the manipulation for the face inversion effect), while in overt word naming, the whole was disturbed while parts (i.e. letters) were kept in their original orientation. The authors however provide no explanation of why they used this other way of experimentally defining orientation sensitivity.

*Thank you for this comment, as we believe this information is important to convey clearly. By not using the same manipulation, we were able to generalize the orientation sensitivity of our groups. Other studies have used alternating font, etc., but we chose the reversed presentation because it was a larger distortion of the whole, as you point out, and would be pushing our hypotheses further. This is now communicated in the text on pg. 10.*

-- Results –

---- Table 1 ----

I suggest including Cohen’s d effect size estimates. Provide units (e.g. ms). I think that at least some of the stats might be wrong. E.g. I calculated Comprehension using https://www.graphpad.com/quickcalcs/ttest2/ I came up with a similar p-value (0.10 instead of 0.09) but a completely different t-value (1.73 compared to 0.51). More generally, please say explicitly in the table heading whether you are presenting regular t-tests or something else in that table.

*Thank you for this comment. Cohen’s d values have been added to Table 1 on pg. 14. Thank you for catching that typo for the t-value for the comprehension measure. We see now that it didn’t make much sense. Yes, I believe the p-value was just a rounding error, as it was .096. It is now reported as .10.*

---- Table 2, table 3, and figure 2 ----

I suggest clearly marking factors as lexical and sublexical as this was a major part of your hypothesis.

*Thank you for this helpful suggestion. It makes a lot of sense for clarity. In the tables (2 & 3), the effects involving lexical factors are now highlighted in light gray, and the sublexical factors have a white background. This is explained in the table footnotes.*

In order to interpret certain factors in the models, the reader needs to know how they were coded. Was e.g. LS 0 and HS 1? Same with others, e.g. presentation orientation.

*Point taken. Yes, LS was coded as 0, and upright/typically presented words were coded as 0. This is now explicitly noted in the text on pg. 14.*

What would help even more with interpretation of these, frankly, quite complicated models with several factors and multiple interactions, is if you would actually present the underlying data graphically. This is done in figure 2 for a few of the variables, but I strongly suggest that you show correlations in a different way that highlights the underlying data points more explicitly (e.g. scatterplots, correlograms: https://www.r-graph-gallery.com/correlogram/). This makes it much easier to compare the groups, and for scatterplots can simultaneously show overall group differences (group main effects) as well as interactions (e.g. to show that the association between orientation and RT is different for the groups). I also suggest that this should not only be shown for the significant interactions, as you expected other differences as well that did not come out as significantly different between the two groups.

*Thank you for this suggestion. We believe it will aid in the transparency in understanding our results. We have included scatterplot figures for the 3-way interactions of the RT data, since that is where our hypotheses were more relevant. We presented the 3-way interactions as two scatterplots, one for typical and one for atypical (reversed) stimuli, with the x-axis representing the psycholinguistic factor (e.g. frequency) and the different colored dots/lines representing the groups. We included these for all psycholinguistic factors, even non-significant ones (see pg. 18).*

---- Principal components analysis ----

The authors rightfully point out that the groups are small for an exploratory factor analysis. I have seen estimates of at least 50 participants, or of at least 10 times the number of participants as there are variables. I found one paper that claims that exploratory factor analysis can be done for very small sample sizes under certain circumstances:

de Winter*, J. D., Dodou*, D. I. M. I. T. R. A., & Wieringa, P. A. (2009). Exploratory factor analysis with small sample sizes. Multivariate behavioral research, 44(2), 147-181.

*Thank you for this. While recognizing the small sample size, we also found this paper helpful and had cited it in the original manuscript (under the Lexical representational structure sub-header on pg. 20, now reference 34).*

However, I think that given the small sample sizes, any differences in factor structure might be uninterpretable. If the authors want to show possible group differences as an exploratory or descriptive analysis, I again suggest that showing two correlograms, one per group, would be more likely to give the reader a gist of what might be going on.

*We apologize. We weren’t quite sure what you were imagining here and are less familiar with correlograms, per se. Since the largest difference in the structure between the two groups was the loading of the phonemic decoding measure, we included a scatterplot of how that correlates with Word ID in both groups. This is now Figure 3. We hope this will highlight the gist of the take-home message here in a clearer manner.*

Reviewer #2: This paper presents the results of a study that examined the existence of different reading styles in English. The majority of studies have been done on reading in English, and the consensual model, the dual route model, holds that written words can be identified via the lexical, or holistic/orthographic route, or a sub-lexical, phonological decoding route. A number of cross language/writing system studies have shown that the orthographic transparency and morphological structure of specific writing systems result in more efficient processing weighting the lexical or the sub-lexical route. In English, it has been shown that both characteristics of the words and of the reader (e.g., skill level) affect the relative use of the lexical or sublexical routes. I have several major concerns:
1. I am not exactly sure of the goal of this paper: participants were divided into a ‘holistic’ or ‘sub-lexical’ category by the degree to which reading words upside-down differed from reading words in the canonical orientation. Then, after a very extensive sifting procedure, these groups were compared on reading words forwards and backwards. I understand that the point was to see if performance on this task, using words for which the holistic and sub-lexical aspects are documented, would go in the same direction. Here lies the problem – the fact that the groups differed on the backwards reading task is trivial, as they were chosen to differ on an upside-down reading task.

*We actually agree here. We reported the results that show group differences in the backwards reading task for the sake of completeness of the model.*

The fact that they only differ on performance on the upside-down words is problematic, as it suggests that in normal word identification tasks, they do not differ. Thus, when the task is made more difficult in a specific manner, then the groups differ in how well they can compensate for the spatial distortion. But that is how the groups were created to begin with – there is no theoretical reason to think that the two spatial manipulations (upside down and backwards) are inherently different.

*Here we respectfully disagree. It is true that the groups only seem to differ when the word presentation is disrupted, but that was actually hypothesized. We interpret these data patterns as suggesting that while both groups are skilled readers, they are actually using different underlying procedures for word identification. Since both groups’ procedures are efficient for them, we wouldn’t necessarily expect differences in a typical word presentation. We predicted that differences would likely emerge when we made the task more difficult, as you stated. However, it isn’t the fact that one group is worse or slower when the words were distorted that we hoped to focus on, because as you stated that was part of the group definitions. We hoped to highlight the fact that different psycholinguistic factors were better predictors of reading speed in the distorted trials. Indeed, we found that high sensitivity readers were significantly more affected by length (and marginally by imageability), both considered lexical factors, when words were distorted, but low sensitivity readers were marginally more affected by biphone frequency, considered to be a sublexical factor. While some of the results are weaker, we hoped to highlight that all observed effects are in the predicted direction, as you state below.*

*To your other point regarding there being no theoretical reason that the two manipulations are inherently different, we actually agree. We wanted to use two different manipulations to strengthen the argument that different manipulations should similarly disrupt reading ability and lessen a potential concern that these effects are specific to one manipulation. We highlight this point on pg. 10.*

2. The finding that the factor structure of the RTs is different in a predictable manner could be even more supporting evidence that this division is reasonable, but the very small number of participants in the groups (11 and 14) really makes it hard to believe it…-- and, it is also true by the way that the groups were defined.

*We hope the response to the above comment will address some of these concerns.*

*Lastly, while indeed some effects are quite small, but in the predicted directions, the 3-way (group x presentation orientation x length) interaction is highly significant (p=.000102), which is very unlikely to be due to chance, and added a reference to support that point on pg. 24 (*Ioannidis JP. The proposal to lower P value thresholds to. 005. Jama. 2018;319(14):1429-30*). We do not believe this effect is directly linked with how the groups were defined, as our criteria for inversion sensitivity did not include length as a factor. But we do agree with your concern about the strength of the other effects. We have tried to be more transparent in the text regarding this issue, both in how we describe the effects, and on page 23 of the discussion, where we explicitly note that some of our results are marginal.*

3. There seem to be some missed opportunities here. Although it is not mentioned, the division of word characteristics into lexical and sub-lexical categories follows results shown by many studies of reading by split-brain patients, reading by unilateral brain damaged patients, and healthy participants identifying words in divided visual field paradigms. This has been done across languages (e.g., Rao & Vaid, 2017; Zhou et al, 2019; Ibrahim & Eviatar, 2012; and many more). Given the very large initial sample, it would have been interesting to see if left-handed participants tended to fall into one or the other group. That would have been a novel result.

*We did not include left-handed subjects in our sample, and so cannot pursue this suggestion. However, we appreciate this thoughtful comment and would be interesting in pursuing these ideas in future work.*

Summary: Other than the very small sample size, the study seems well done. I am just not sure what it adds to the literature.

Reviewer #3: The investigators have concluded that their results revealed that greater orientation sensitivity was associated with a reading profile that relies less on sublexical phonological measures and more on lexical-level characteristics within the skilled English readers. This is based on a statistical approach involving typical procedures such as the t-test, mixed models, the empirical logit model and principal component analysis. There are some concerns. Specifically:

1. The authors note that participants were recruited from a database of 411 individuals interested in study opportunities. It is not clear what ‘study opportunities’ means here or if it is even relevant.

*We apologize for any unwanted ambiguity. The Perfetti Lab recruits several hundred participants each year for a behavioral screening in order to drive several more specific research studies where participants are needed with specific behavioral characteristics. We were conducting one of those many studies.*

They explain that a number of subjects were removed leaving 203 from an initial 411 potential participants. This appears to be a convenience sample.

*We first filtered based on overall reading ability, in the hopes to avoid testing participants at the extremes of the distribution. We’re not sure this would necessarily be considered convenience sampling, which to our understanding would be recruiting only participants that were available or nearby.*

A hypothesis is stated on page 7 in the form that native English readers who show more sensitivity to atypical orientation do so because they make greater use of holistic orthographic coding, which should be reflected in a distinctive reading profile. This appears to be a comparison of the low to high sensitivity group which they define. The problem is that there is no statistical design motivation for the sample size. Is 203 individuals statistically sufficient to test their hypothesis with reasonable statistical power?

Also on the bottom of Page 8 the investigators note that participant attrition, due to graduation since participation in initial screening and eligibility requirements for a parallel imaging study (beyond the scope of the current study), also reduced the potential participant pool. What exactly does this mean and what was the final number of participants for each of the Tables 1 to 5?

*That is correct. There were several extenuating circumstances that lead to a smaller sample including that we wanted to first recruit participants with nonoverlapping inversion sensitivity scores (described and highlighted on page 9), and that they also be eligible for a companion imaging study. Along with attrition from the original database due to graduation or simply not responding, the pool that we drew from was significantly smaller. We believe future research will be important to understand how common these behavioral profiles are.*

*The final number of participants for all tables was 14 for LS readers and 11 for HS readers.*

2. As minor points please define RT on page 8. It appears to be 'reaction time'. On the top line of page 20 the term ‘ phonological decoding (.728)’ should be ‘Phonemic Decoding (0.728)’ as per Table 5.

*Thank you- these corrections have been made.*
