# Supplementary material for: Unmasking individual differences in adult reading procedures by disrupting holistic orthographic perception
Source: PLoS One. 2020 May 26;15(5):e0233041. doi: 10.1371/journal.pone.0233041 (PMC7250424; doi:10.1371/journal.pone.0233041)
Supplement: Attachment — Submitted filename: PLoSOne_SecondRevision_ResponseToReviewers.docx [file pone.0233041.s004.docx]

Dear Dr. Yafit Gabay and Reviewers,

First, we apologize for the delay in response as we adjusted to impending and current changes in daily life. We very much appreciate the opportunity to address the questions from past and new reviewers and believe they have greatly improved the paper again. Responses to individual comments are below in blue italics. Many thanks to all, and we hope everyone is safe and healthy.

Sincerely,

Elizabeth Hirshorn, PhD

**Reviewer #1:**

1. Regarding the comment of reviewer #2: „...the fact that the groups differed on the backwards reading task is trivial, as they were chosen to differ on an upside-down reading task.“ I started looking into this, and what I missed in my first review of the paper is that the authors apparently not only assessed the orientation sensitivity for the second time, but they also redefined their groups based on this dependent measure: „Six participants whose overt naming scores were neither above nor below the median orientation sensitivity that was consistent with their initial group assignment based on the lexical decision task were removed.“ I don‘t think that this is a good idea, if I understand correctly what the authors did, as this can be considered double dipping into the data. As this was done, I don‘t think that the main effects of orientation or interactions with orientation should be trusted, although I suggest getting a second opinion from a statistician. I suggest rerunning all models with these participants included. Otherwise, group assignments and orientation effects are not independently assessed.

***Thank you for this comment and the chance to consider this concern. We understand the concern of ‘double dipping,’ and agree that the 2-way interaction between group and orientation is a trivial finding, since that is essentially how the groups were defined. We consulted with a statistician, who confirmed that ‘double-dipping’ should not affect the direction or strength of the 3-way interactions, since there is not anything inherent about being sensitive to inversion, per se, that would cause one group to be more influenced than another by a psycholinguistic measure when words were presented atypically. In fact, that is what we were interested in testing. Furthermore, words that were presented in the typical and atypical orientations were matched for all psycholinguistic factors (e.g., there weren’t more long words that were atypical than typical).***

***Nevertheless, we did take your advice and added in the previously removed participants and ran the model again with “group” as a continuous variable based solely on the lexical decision task (LDT), which eliminates the double-dipping component of group selection since it doesn’t incorporate the naming task at all. The 3-way interaction between “group” (i.e., strength of inversion sensitivity in LDT task) x presentation orientation x length was actually even more significant with this approach (see Table below- yellow highlighted row).***

***Specifically, the previous t-value for that 3-way interaction was 3.89, and adding the other participants led to a t-value of 6.12. We believe this provides further support that the group/sensitivity x orientation x length 3-way interaction is robust. The two marginal 3-way interactions did not hold up (see response about power for Reviewer #4 below), but another marginal 3-way interaction emerged in the predicted direction, such that bigram frequency was a marginally better predictor of RT for atypically presented words in those whose LDT were relatively small (i.e., those with low sensitivity). We previously discussed our marginal effects in a tentative manner, so this change in marginal results does not undermine our results. Instead, we believe that the fact that a conceptually related marginal effect emerges supports the same weak overall pattern of psycholinguistic differences between the two groups.***

***Despite the strengthening of the group x orientation x length effect in the updated model, we are proposing to keep the main reporting of results the same, as a group design, but propose to add the results of the additional analysis for transparency (see pg. 17-18). We propose to keep the main focus on the original group-based analysis for the following reasons:***

- ***Our a-priori hypotheses and design were meant for having separate groups.***
- ***Related to reviewer #4’s concern that inverting words alone may not be enough to measure ‘holistic processing,’ we believe that using two similar, but not identical measures actually strengthens different aspects of the study.***
  - ***For example, based on logistics and timing, the LDT experiment did not have a lot of trials and we wanted to reduce any potential noise by combining it with an additional task that was different, but conceptually similar in order to have more stability in our measurement.***
  - ***We recently published a paper (Carlos et al, 2019) that used this same grouping. In this paper the same Ss were used to look at group differences in a neural measure of lateralization, with more inversion sensitive participants exhibiting a more bilateral pattern of activation in a putative visual word form area. This result provides converging evidence that our groups exhibit different patterns of orthographic processing, with no concerns about “double-dipping” into the same data.***
  - ***Since these two papers are in essence companion pieces, we believe it is more straightforward to be able to talk about the same participants.***

***That being said, our current and future plans for the continuation of this research will be using more continuous measures of sensitivity.***

What is going on in the new figure 2 in terms of RTs of atypical word presentations? Almost all of the RTs for the low sensitivity (red) seem to fall under the red regression line while almost all of the RTs for the high sensitivity (cyan) seem to fall under the cyan regression line. I suspect that this has something to do with the actual drawing of the graph, i.e. perhaps almost all of the red dots on the top are underneath the cyan dots on the top. This could likely be amended by making them partially transparent.

***Thank you for sharing this observation. The pattern that you noticed could be partially a consequence of the group difference in RT for atypically presented words (low sensitivity readers are faster than high sensitivity readers by design), but we understand what you’re saying. At your suggestion, we did make a version with more transparent markers, but unfortunately it just ended up looking extremely blurry and harder to interpret. For the sake of perceptual clarity, we think the information is better represented with more opaque markers, albeit less than ideal. We believe that the main take-away message from this figure, to see the non-parallel lines in the atypical presentation for length (plus imageability & biphone frequency) should still be able to be communicated.***

2. Also, the x-axis of figure 2 just says value.z. I suggest changing this to something a bit more transparent, and saying in the figure legend that the x-axis represents each psycholinguistic factor.

***Thank you for this suggestion. This was changed, so that the x-axis is now titled, ‘z-score of Each Psycholinguistic Factor,’ thank you.***

3. Finally, for that graph, please say explicitly that these are (if they are) RTs from individual trials pooled across participants.

***Thank you for this suggestion. This information was added to the figure caption.***

4. The statistics in Table 1 still look weird. The t-value(s) and p-value(s) still don‘t match. I am assuming an N of 14 LS and 11 HS.

***Thank you for pointing this out. We regret that the previous version did indeed have errors in Table 1. The small edits needed to correct these errors have been made. Importantly, the results are essentially the same.***

**Reviewer #4**

The authors show that greater sensitivity to orientation/greater holistic processing is associated more with lexical than sub-lexical characteristics, thus holistic strategies seem to be involved with lexical reading strategies. I was not one of the three original reviewers. My fellow colleagues did an excellent job in their revisions and I also consider the response of the authors adequate. I have only a few points that the authors might additional take into consideration.

1. One question is the very small number of participants. I wonder if the authors could provide a power analysis study using e.g., g*power.

***We understand the concern about a small sample. We consulted with a statistician, and instead of using g*power, we used a function in R that is suited to linear mixed models (see powerSim in simr package). This allows us to estimate the power for specific effects in our model using Monte Carlo estimation:***

***Green, P., & MacLeod, C. J. (2016). SIMR: an R package for power analysis of generalized linear mixed models by simulation. Methods in Ecology and Evolution, 7(4), 493-498.***

***Brysbaert, M., & Stevens, M. (2018). Power analysis and effect size in mixed effects models: A tutorial. Journal of Cognition, 1(1).***

***We ran this for each of the three 3-way interactions that showed effects, set at 200 simulations. The 3-way interaction between group x orientation x length had a very large effect size, and the observed power was 96.50% (95% confidence interval: 92.92, 98.58). Not surprisingly, the two marginal effects had much lower power- both had 51% power (43.85, 58.12). We hope that talking about these effects as more tentative will be adequate.***

***We have modified the manuscript to include this power analysis in the results section (pg. 17), and have retained our previous caution in presenting and discussing our marginal effects.***

2. An indicator of holistic word processing—observers’ sensitivity to changes in configural/spatial jittering information of objects in an inversion paradigm has been used by Wong et al. (2019). Is this manipulation closer to what is meant by holistic processing? Just inverting the stimuli maybe not be enough to study holistic processing.

***Thank you for this comment, as this is an ongoing issue in the field. In fact, since this work has been partially inspired by the face processing literature, we share the concern that there is not necessarily one manipulation that completely encompasses the construct of ‘holistic’ processing (see Rezlescu et al, 2017). That is one of the main reasons we chose to combine slightly different measures for group assignment.***

***Rezlescu, C., Susilo, T., Wilmer, J. B., & Caramazza, A. (2017). The inversion, part-whole, and composite effects reflect distinct perceptual mechanisms with varied relationships to face recognition. Journal of Experimental Psychology: Human Perception and Performance, 43(12), 1961.***

***That being said, the current and future work in this research program uses a combination of measures, including a composite task. For this paper, we have added a paragraph to our discussion section (pg. 28) that notes this important issue, with citations to Wong et al. (2109) and Rezlescu et al. (2017) included.***

3. Sensitivity to atypical orientation in the overt naming task used half of the stimuli presented in typical orientation and half presented in a reversed (FLIGHT à THGILF) orientation. What has this to do with holistic strategy?

***Our thinking is that any perceptual manipulation that disrupts the typical processing of an object should also disrupt holistic processing, if that is used. For example, other studies have used case, size, and font alteration to add visual noise and thus disrupt ‘holistic’ processing (see Pae et al, 2017).***

***Pae, H. K., Kim, S. A., Mano, Q. R., & Kwon, Y. J. (2017). Sublexical and lexical processing of the English orthography among native speakers of Chinese and Korean. Reading and Writing, 30(1), 1-24.***

***While our reverse presentation is not a widely used manipulation, we consider it to be a conceptual extension of previous manipulations (such as case alternation) – that is, the idea that presenting a word in any atypical orientation should disproportionately disrupt those who rely more on a ‘holistic’ reading procedure.***

***We note that similar ideas are present in the face and object processing literature. For instance, Perret, Oram, and Ashbridge (1998) propose that the speed of object recognition rests upon the accumulation of activity from neurons selective for the object as experienced in a particular viewing circumstance. This neuronal tuning is presumed to be sensitive to frequency of occurrence, and so the neural activity builds more quickly for objects presented in a canonical as compared to unusual view.***

4. Authors hypothesize that the HS group should be more affected than the LS group by lexical-level factors when words are atypically oriented, whereas LS should be more affected than HS by sublexical-level factors when words are atypically oriented. But is it equally possible to make the same type of predictions for when words are typically oriented and lexical factors more at play?

***This is a very astute point. The reason we focused on atypically oriented words is that the effects may be smaller and not as easily measured when words are typically oriented, since both groups are skilled and efficient using whatever reading procedures are most natural. In contrast, with an atypical orientation the idea is that all participants are forced to rely more upon sublexical procedures, and so this helps to “unmask” differences in the ability to use these procedures.***

***However, we do agree that biases for the different procedures remain present when words are presented in their typical orientation. In support of this point, in a previous paper, we compared Chinese- and Korean-English bilinguals:***

***Ben-Yehudah, G., Hirshorn, E. A., Simcox, T., Perfetti, C. A., & Fiez, J. A. (2019). Chinese-English bilinguals transfer L1 lexical reading procedures and holistic orthographic coding to L2 English. Journal of Neurolinguistics, 50, 136-148.***

***As we discuss in the introductory section of our manuscript, in this prior work we tested our prediction that Chinese-English bilinguals would use a more holistic reading procedure than Korean-English bilinguals. In support of this hypothesis, we found that the Chinese-English bilinguals exhibited greater sensitivity to inversion than the Korean-English bilinguals.***

***One finding from this paper that we did not previously discuss concerned differences in the reading skill profile of the two bilingual groups. Even though we matched the groups on the basis of spoken English experience and ability, we found that the Chinese-English bilinguals exhibited performance differences on typically presented items. For instance, they performed more poorly on the Word Attack and Word ID subtests of the Woodcock Reading Mastery Tests, and we found that the Chinese-English bilinguals exhibited greater sensitivity to lexical frequency on a word-naming task that included upright and inverted items. In the discussion section of Ben-Yehudah et al. paper, we discuss the implication of these results, which are similar to those reported in previous studies. Specifically, we suggest that Chinese-English bilinguals have a bias towards a holistic procedure is a less optimal approach for developing reading skill in an alphabetic writing system. Consequently, they require more reading experience to attain the same level of skill****.*

***These same ideas could be relevant for native English speakers to exhibit a bias towards a holistic procedure. However, we would be unlikely to find them in the present study, because we selected the two groups to be matched on measures of reading skill. If we instead could have selected the group to be matched on reading experience or randomly selected them without concern for reading skill, then it is very possible that we would have observed the pattern suggested by the reviewer.***

***Due to concerns about the overall length of the paper, we have not revised our discussion section to consider this point. However, if the reviewer feels that this is an issue that merits consideration, we would be pleased to add text addressing the reviewer’s comment.***
